# Case report: Multi-organ injuries induced by tislelizumab

**DOI:** 10.3389/fimmu.2025.1508293

**Published:** 2025-02-04

**Authors:** Man Yuan, Ning Han, Li Shu, Libo Yan, Hong Tang

**Affiliations:** ^1^ Center of Infectious Diseases, West China Hospital of Sichuan University, Chengdu, China; ^2^ Division of Infectious Diseases, State Key Laboratory of Biotherapy, Sichuan University, Chengdu, China; ^3^ Department of Infectious Diseases, Suining Central Hospital, Suining, China

**Keywords:** lung squamous cell carcinoma, immune-related adverse events, immune checkpoint inhibitors, tislelizumab, multi-organ injuries, case report

## Abstract

The use of immune checkpoint inhibitors (ICIs) often develops immune-related adverse events (irAEs). However, irAEs-induced multi-organ injuries remain a rare event. We herein report a case of multi-organ injuries induced by tislelizumab in a lung squamous cell carcinoma (LUSC) patient. A 68-year-old man had undergone neoadjuvant chemotherapy with paclitaxel, carboplatin, and tislelizumab. He presented with a 1-month history of nausea and poor appetite after the second dose of therapy. During investigations, rhabdomyolysis, liver, kidney, and thyroid damage were detected. After multi-disciplinary consultation, multi-organ injuries related to ICIs (striated muscle, liver, kidney, and thyroid) were considered to result from cumulated irAEs induced by tislelizumab. The patient was treated with levothyroxine, methylprednisolone, intravenous immunoglobulins, and continuous renal replacement therapy. After treatment, the patient recovered and was discharged from the hospital. The patient presented with multiple organ damage, not single immunity treatment adverse reactions, relatively rare. In clinical work, irAEs are likely not a single-system organ disorder and many kinds of attention need to be combined with the risk of multi-system damage.

## Introduction

Recent studies have focused on immune checkpoint inhibitors (ICIs). In patients with unresectable tumors, ICIs are used as the first-line therapy in a variety of fields, which has contributed to substantially improved survival. The use of these ICIs often develops immune-related adverse events (irAEs), which are becoming essential safety issues worthy of attention despite the exciting therapeutic prospects. Multi-organ injuries associated with the use of ICIs are rare compared with other irAEs.

Tislelizumab, a humanized IgG4 anti-programmed cell death protein-1 (PD-1) monoclonal antibody, was launched in China in December 2019. It prevents autoimmune responses by promoting the apoptosis of antigen-specific T cells in lymph nodes while reducing the apoptosis of regulatory T cells (1). Tislelizumab, in combination with paclitaxel and carboplatin, was approved as the first-line treatment option in patients with advanced squamous non-small-cell lung cancer in China in 2021 (2). A few adverse reactions to tislelizumab have been reported. The common adverse reactions include fatigue, rash, hypothyroidism, and elevated aminotransferase. We herein report a case of tislelizumab-associated rhabdomyolysis, liver, kidney, and thyroid damage in a lung squamous cell carcinoma (LUSC) patient. We present this case in accordance with the CARE reporting checklist.

## Case description

**Figure 1:**
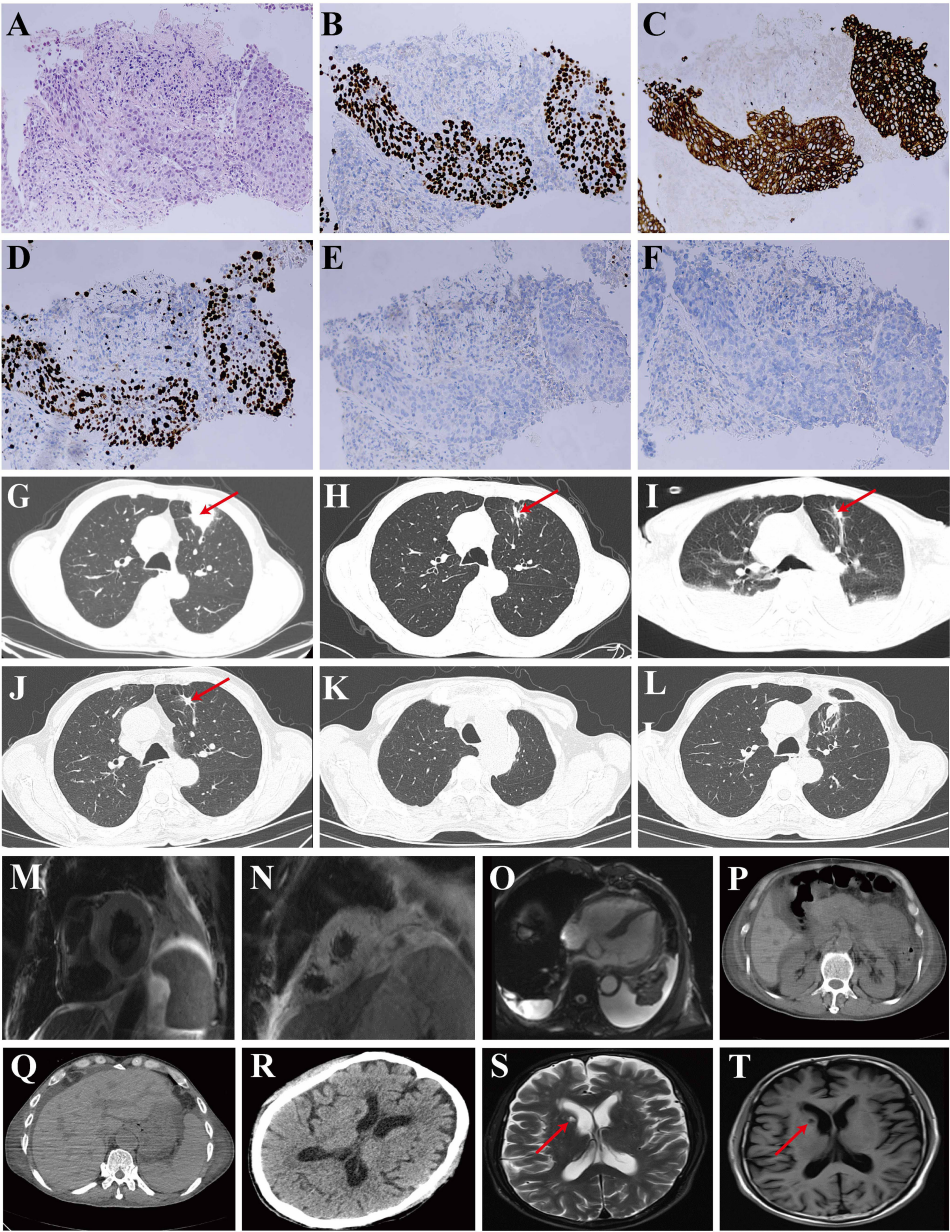
Pathological images of the lung biopsy and imageological examination. **(A)** Hematoxylin and Eosin (HE) staining; **(B)** P40 immunohistochemical staining; **(C)** Pan-cytokeratin (AE1/AE3) staining; **(D)** Ki-67 proliferation index showing expression levels up to 60% in tumor cells; **(E)** Thyroid Transcription Factor-1 (TTF-1) staining; **(F)** Napsin A staining. **(G-L)** Chest computed tomography (CT) scans: **(G)** Image acquired on December 5, 2023, revealing a soft tissue density shadow in the anterior segment of the left upper lobe, measuring approximately 3.4 x 3.1 cm (indicated by a red arrow); **(H)** Image acquired on February 20, 2024, demonstrating shrinkage of the soft tissue (indicated by a red arrow); **(I)** Image acquired on April 15, 2024, showing a small mass measuring 1.4 x 0.9 cm (indicated by a red arrow) in the anterior lobe of the left superior lung, with evidence of bronchial wall truncation, peripheral pleural stretch and bilateral pleural effusions; **(J)** Image acquired on July 9, 2024, pre-operatively; **(K, L)** Images acquired on August 27, 2024, post-operatively. **(M-O)** Cardiac MRI. **(P, Q)** Abdominal CT. **(R)** Head CT revealed encephalatrophy and demyelination in the white matter. **(S, T)** Head MRI. The brain MRI indicated old infarcts in the lateral ventricle, as denoted by the red arrow.

**Figure 2:**
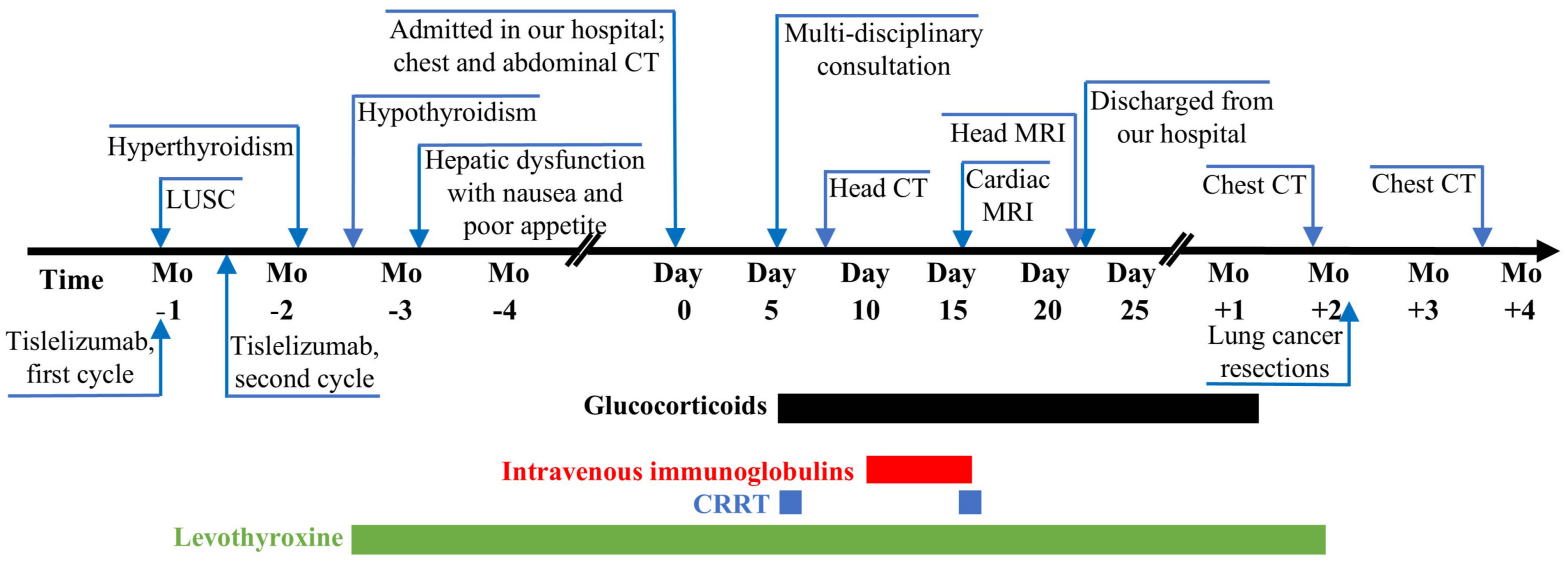
Clinical course of the diagnosis and treatment.

The patient was a 68-year-old man with a smoking history of 40 years. He had been diagnosed as having LUSC (cT3N0M0 IIB) by percutaneous needle lung biopsy in December 2023 at the local hospital. The pathology report showed P40 (+), pan-CK (AE1/AE3) (+), TTF-1 (-), NapsinA (-), CgA (-), CD56 (-), and Ki-67 (60% +) (**Figures 1A–F**). A contrast-enhanced chest computed tomography (CT) scan revealed a subpleural soft tissue density shadow in the anterior segment of the left upper lobe, with a maximum crosssectional dimension of approximately 3.4 × 3.1 cm (**Figure 1G**). Then he had undergone neoadjuvant chemotherapy with paclitaxel (400 mg d1), carboplatin (400 mg d1), and tislelizumab (200 mg d1; BeiGene, China) Q3W in December 2023 at the local hospital. The first session of treatment went well, with no significant adverse effects. After the second dose of treatment in February 2024, he developed hyperthyroidism and then hypothyroidism and was given metoprolol succinate followed by levothyroxine. A follow-up CT scan showed significant reduction in the mass size (**Figure 1H**). In March 2024, he experienced nausea, poor appetite, and abnormal liver function markers and was admitted to West China Hospital of Sichuan University in April 2024. The time axis of diagnosis and treatment of the patient is shown in **Figure 2**. Since November 2023, he had been on atorvastatin calcium and aspirin for cerebral infarction without limb movement issues. He stopped atorvastatin in March 2024 due to liver function abnormalities.

On evaluation at our institution, his body temperature was 36.5°C, with a pulse of 110 beats per minute, blood pressure of 98/72 mmHg, and a breath of 20 times/min. Hematology showed a hemoglobin level of 88 g/L, a white blood cell count of 7.06×10^9^/L, 89.2% neutrophils, and 6.9% lymphocytes. Chest CT revealed a small mass (1.4 × 0.9 cm) in the anterior lobe of the left superior lung, bronchial wall truncation, and peripheral pleural stretch (**Figure 1I**).

He had conjunctival icterus. Laboratory evaluation showed significantly deranged liver and renal function (**Table 1**). Evaluation for other causes was conducted. Pathogen investigations, including hepatitis A, B, C, and E, syphilis, human immunodeficiency virus, cytomegalovirus, Epstein-Barr virus serum, serum (1,3)-beta-D-glucan, serum galactomannan, and sputum culture were all negative. Additional autoimmune disease markers were also investigated. Antinuclear antibodies were 1:100 positive. Anti-neutrophil cytoplasmic antibody was within normal range. Urinalysis showed haematuria (24 red blood cells/high-power field) and proteinuria (1+). Renal ultrasound showed no issues. Abdominal CT indicated a slight decrease in liver density, with normal kidney results (**Figures 1P, Q**).

He had conjunctival icterus. Laboratory evaluation showed significantly deranged liver and renal function (**Table 1**). Evaluation for other causes was conducted. Pathogen investigations, including hepatitis A, B, C, and E, syphilis, human immunodeficiency virus, cytomegalovirus, Epstein-Barr virus serum, serum (1,3)-beta-D-glucan, serum galactomannan, and sputum culture were all negative. Additional autoimmune disease markers were also investigated. Antinuclear antibodies were 1:100 positive. Anti-neutrophil cytoplasmic antibody was within normal range. Urinalysis showed haematuria (24 red blood cells/high-power field) and proteinuria (1+). Renal ultrasound showed no issues. Abdominal CT indicated a slight decrease in liver density, with normal kidney results (**Figures 1P, Q**).

**Table 1:** Laboratory values.

Laboratory values	Normal range	Before admission			Days after admission								Months after discharge				
		Dec-23	Jan-24	Feb-24	1	3	5	8	10	15	20	23	0.5	1	2	3	4
TBiL (μmol/L)	3.2-20.5	7.7	13.3	–	297.7	283.8	185.2	108.3	84.4	58.3	51.5	39.1	34.1	22.2	–	24.6	7.6
DBiL (μmol/L)	<6.8	3.5	10.6	–	254.2	245.6	159.7	95.1	75.3	52	44.4	33.8	21.8	5.5	–	4.7	1.3
TBA (μmol/L)	<15	–	–	–	222.8	58.7	113.5	124.4	51	23.7	15.9	9.9	–	–	–	–	–
ALT (U/L)	<50	10	28	–	61	55	36	72	69	110	150	135	37	17	–	94	15
AST (U/L)	<40	17	24	–	311	304	179	162	84	120	115	89	24	20	–	88	28
ALP (U/L)	45-125	77	85	–	533	450	333	231	206	202	185	159	101	86	–	–	82
GGT (U/L)	<60	23	27	–	249	234	210	116	102	84	83	76	63	46	–	–	34
ALB (g/L)	40-55	38.5	46.1	–	25.7	27.5	27.4	36.3	37.1	31.6	29.6	32	36.2	37.1	–	–	29.8
GLB (g/L)	20-40	24.6	25.8	–	13.7	13.2	13.7	14.8	10.8	23	19.5	17.8	17.2	21.3	–	–	17.1
CREA (μmol/L)	68-108	88	83.4	–	405	432	421	315	288	145	104	89	75.2	86.6	–	94	79.5
CK (U/L)	50-310	–	–	–	1553	1150	1516	–	346	1005	738	559	22	–	–	–	–
LDH (U/L)	120-250	–	–	–	480	457	447	–	523	563	557	528	215	–	–	–	–
BNP (ng/L)	<486	–	–	–	3884	9550	6547	5258	–	1521	1087	1022	–	–	–	24.2	–
Myo (ng/mL)	<72	40.6	–	–	>3000	>3000	>3000	>3000	–	>3000	>3000	>3000	–	–	–	–	–
CK-MB (ng/ml)	<6.22	2.51	–	–	18.21	7.92	8.56	7.71	–	–	13	8.22	–	–	–	–	–
TPN-T (ng/L)	<14	0.02	–	–	43.2	44.3	86.8	82.1	–	103	104	115	–	–	–	–	–
TSH (mIU/L)	0.27-4.2	2.02	–	0.02	41.2	–	–	–	–	–	–	–	47.4	23.9	1.28	0.27	0.07
FT3 (pmol/L)	3.60-7.50	4.73	–	27.7	4.17	–	–	–	–	–	–	–	3.05	3.84	6.50	6.74	5.72
FT4 (pmol/L)	12.0-22.0	12.34	–	54.4	4.74	–	–	–	–	–	–	–	12.5	14.9	18.3	21.6	17.1

TBiL, total bilirubin; DBiL, direct bilirubin; ALT, alanine transaminase; TBA, total bile acid; AST, aspartate transaminase; ALP, alkaline phosphatase; GGT, gamma-glutamyl transferase; ALB, albumin; GLB, globulin; CREA, creatinine; CK, creatine kinase; LDH, lactate dehydrogenase; BNP, B-type natriuretic peptide precursor; Myo, myohemoglobin; CK-MB, creatine kinase-myocardial band; TPN-T, troponin-T.

The muscle strength of the upper limbs was 3, and the lower limbs were 2. The ability of the patient to perform daily physical activities was limited, especially in the lower limbs. Serum levels of creatine kinase (CK) and lactate dehydrogenase (LDH) had increased (**Table 1**). Head CT revealed no new cerebral hemorrhage, and head magnetic resonance imaging (MRI) showed no new infarcts (**Figures 1R-T**). Rhabdomyolysis was considered based on the clinical manifestation above.

Moreover, cardiac damage parameters were altered with elevated levels of myohemoglobin, troponin-T, creatine kinase-myocardial band (CK-MB), and B-type natriuretic peptide precursor (BNP) (**Table 1**). An electrocardiogram (ECG) showed paroxysmal atrial fibrillation. Echocardiography showed a normal left ventricular ejection fraction. Cardiac MRI indicates cardiac enlargement and interventricular septal thickening, with no significant abnormalities in myocardial signal or apparent dysfunction in the ventricles (**Figures 1M-O**).

Thyroid function evaluation showed a concentration of thyroid-stimulating hormone 41.2 mIU/L [normal range 0.27-4.2], free triiodothyronine 1.47 pmol/L [normal range 3.60-7.50], free thyroxine 4.74 pmol/L [normal range 12-22], suggesting hypothyroidism.

After multi-disciplinary consultation, given the absence of underlying cardiovascular diseases, evidence of virus or other pathogen infections, and a history of ICIs administration before hospitalization, as well as multiple organ involvement simultaneously, multi-organ injuries related to ICIs (striated muscle, liver, kidney, and thyroid) were considered to result from cumulated irAEs induced by tislelizumab. Then the patient received methylprednisolone (80 mg daily for nine days, tapering gradually after two months), intravenous immunoglobulins (IVIG) (15 g daily for four days), and continuous renal replacement therapy (CRRT), with improvement leading to recovery, and was discharged from our hospital in May 2024 (**Figure 2**). On follow-up in the outpatient, he had a lung cancer resection at the local hospital in July 2024. Postoperative chest CT found no metastasis (**Figure 1J**, pre-operative; **Figures 1K, L**, one month postoperatively).

## Discussion

In patients with LUSC undergoing treatment with ICIs, the most frequently observed irAEs are asthenia (10%), decreased appetite (11-19%), dermatologic toxicity (4-28.6%), diarrhea or colitis (8-11.4%), fatigue (16-33%), nausea (8.6-15%), and pyrexia (5-14.3%) (3). Severe adverse events, classified as Grade 3 or higher, are rare (3). This case report described a LUSC patient with multi-organ injuries (striated muscle, liver, kidney, and thyroid) induced by tislelizumab. To our knowledge, this is the first report of tislelizumab-associated multi-organ injuries with rhabdomyolysis, liver, kidney, and thyroid damage. The patient also received paclitaxel and carboplatin chemotherapy. Common side effects of paclitaxel include hypersensitivity, myelosuppression, bradycardia, hypotension, peripheral neuropathy, muscle and joint pain, nausea, diarrhea, mouth sores, and hair loss (4). Carboplatin’s main dose-limiting side effects are myelosuppression (thrombocytopenia) and peripheral neurotoxicity (5). Paclitaxel and carboplatin rarely cause cholestatic liver injury. Paclitaxel can elevate serum aminotransferase levels in 7% to 26% of patients, but levels exceed 5 times the upper limit of normal (ULN) in only 2% of high-dose cases (6). Similar rates of alkaline phosphatase (ALP) elevation and occasional mild bilirubin increases are observed (6). Paclitaxel is not convincingly linked to delayed, idiosyncratic liver injury with jaundice (6). While up to one-third of patients on carboplatin may experience mild, temporary increases in serum aminotransferase levels, significant liver injury from carboplatin is very rare and not well characterized (7). Carboplatin and pemetrexed usually present a low to moderate risk of transaminitis, typically without causing substantial liver injury or jaundice (8). Diagnosing drug-induced liver injury (DILI) can be customized based on the liver injury pattern, using the R-value: serum alanine aminotransferase (ALT)/ULN divided by ALP/ULN (9). This categorizes the injury as hepatocellular (R > 5), mixed (R = 2-5), or cholestatic (R < 2). To our knowledge, only one case of a patient with esophageal and thoracic squamous cell carcinoma receiving radiotherapy and concurrent chemotherapy with paclitaxel and cisplatin has been reported to develop cholestatic DILI (10). Checkpoint inhibitor-induced liver injury (CHILI), occurring in up to 25% of patients, is primarily treated with steroids (11). Lina Hountondji et al. conducted a multicenter cohort study with 117 patients to describe the clinical patterns of CHILI (12). CHILI was categorized as cholestatic (36.8%), hepatocellular (38.5%), or mixed (24.8%), with cholestatic and hepatocellular patterns being the most common. Steroid treatment was given to 79.5% of patients. Kazuyuki Mizuno et al. found a cholestatic pattern in 58.6% (17/29) of CHILI cases (13). Our patient showed a cholestatic liver injury pattern, marked by high ALP levels (R < 2) and jaundice. Abdominal ultrasound and CT ruled out biliary obstruction, aligning more closely with ICI-induced liver injury. Recently, numerous guidelines have been developed for managing irAEs (11, 14–16). Treatment recommendations depend on the affected organ(s), CTCAE grade, and patient comorbidities. Immunosuppression, primarily with high-dose corticosteroids, is the cornerstone of treatment. For severe toxicities, guidelines advise tapering corticosteroids over 4-6 weeks, resulting in extended immunosuppression. Subsequently, treatment with methylprednisolone confirmed the cause, leading to near-complete liver recovery in a month. Kidney issues were evident through proteinuria, hematuria, and a rise in serum creatinine. High CK, LDH, and aspartate transaminase (AST)/ALT ratios, along with reduced muscle strength, indicated rhabdomyolysis. Rhabdomyolysis due to paclitaxel/carboplatin is rare, with only one reported case of myositis after paclitaxel-based chemotherapy in an HIV-negative Kaposi’s sarcoma patient (17). Other instances involve high doses of paclitaxel/ifosfamide/carboplatin/etoposide or carboplatin/etoposide/ifosfamide (18, 19). Our case involved standard doses of paclitaxel and carboplatin, making rhabdomyolysis unlikely. While rhabdomyolysis is associated with the development of acute kidney injury, the risk remains low in non-traumatic instances when serum CK levels are below 15,000-20,000 U/L (20). In this case, the patient exhibited an increase in CK levels, reaching up to 1,553 U/L, which may suggest a reduced likelihood of acute kidney injury secondary to rhabdomyolysis.

The adverse effects associated with statin therapy encompass cramps, myalgia, weakness, and, less frequently, rhabdomyolysis (21). The administration of statins in individuals with hypothyroidism poses substantial risks. It is probable that these mechanisms interact synergistically in hypothyroid patients receiving statins, increasing the likelihood of myopathy, particularly at elevated statin dosages (22). To date, there have been reports of rhabdomyolysis occurring in cases of undiagnosed hypothyroidism (20). These patients exhibited muscle symptoms within days to weeks following the initiation of statin therapy. The symptoms resolved upon the discontinuation of statins and the correction of hypothyroidism through thyroxine replacement (20). The normalization of CK levels after hypothyroidism treatment confirmed that hypothyroidism was the primary cause of rhabdomyolysis (20). In our case, the patients were prescribed atorvastatin calcium on November 2023, during which liver, renal, and thyroid functions remained stable. Following the administration of a second dose of tislelizumab on February 24, 2024, the patient developed hypothyroidism and was subsequently prescribed levothyroxine. In March 2024, the patient discontinued statin therapy due to abnormalities in liver function. By April 2024, the patient exhibited CK abnormalities. The persistence of abnormal CK levels, despite more than two months of levothyroxine treatment and over a month since the discontinuation of statins, indicated that statins were not the causative factor for rhabdomyolysis in this hypothyroid patient.

Among irAEs, ICIs-induced cardiovascular toxicity remains a significant concern. Cardiovascular toxicities can present as myocarditis, complete heart block, atrial fibrillation, ventricular arrhythmia, and heart failure (23). Diagnosing ICIs-related myocarditis is complex, requiring a thorough evaluation of clinical symptoms and test results. Diagnosis primarily relies on cardiac biomarkers (such as troponin, BNP, ECG, echocardiography, cardiac MRI, and, when necessary, myocardial biopsy (24). As a gold standard for diagnosis, myocardial biopsy is limited due to its invasive nature. Despite elevated myohemoglobin, troponin-T, CK-MB, and BNP levels, our case report debates the extent of cardiac involvement. A CK-MB to CK ratio exceeding 6% is considered specific for myocardial injury, whereas a ratio below 6% is indicative of skeletal muscle damage or non-cardiac etiologies (25). In the present case, the CK-MB to CK ratio was observed to be less than 2%, suggesting that the elevation in CK-MB is likely secondary to rhabdomyolysis. Furthermore, troponin-T levels also appear to be elevated in a nonspecific manner. The elevation in BNP is potentially attributable to atrial fibrillation. Additionally, it is noteworthy that BNP levels may also be elevated in numerous cancer patients due to inflammation associated with the malignancy itself (24). Given the patient’s compromised general condition, a cardiac MRI was performed on the 16th day post-admission, following more than 11 days of steroid therapy. The administration of steroids may have partially influenced the myocardial damage detected by the MRI. Therefore, myocardial involvement was not well defined.

In this case, the patient initially presented with hyperthyroidism, which subsequently progressed to persistent hypothyroidism. Endocrine events are among the most prevalent toxicities associated with ICIs, affecting up to 40% of patients undergoing treatment (26). The thyroid gland is the most frequently affected endocrine organ by ICIs, typically presenting as hypothyroidism, which may be preceded by transient thyrotoxicosis due to thyroiditis (26). In certain cases, patients may develop persistent primary hypothyroidism (27). The underlying pathophysiological mechanism is believed to involve immune-mediated acute inflammation, leading to the destruction of the thyroid gland (28). The activation of T cells, along with the involvement of various antibodies and cytokines, plays a crucial role in both the initiation and progression of the disease (28). The patient had abnormal thyroid function in the early stage. Thyroid damage may serve as an indicator of potential multi-organ injuries, necessitating heightened vigilance among physicians for complications in other organs both at the time of diagnosis and during follow-up.

ICIs have transformed the treatment landscape for a variety of solid tumors and hematological malignancies, thereby increasing the benefits for more cancer patients. However, an increasing number of adverse reactions caused by ICIs have also been reported. ICIs can induce irAEs, and up to 70% of patients undergoing ICIs therapy can have irAEs (29). These toxic effects are thought to be mediated by autoreactive T cells no longer kept in check by feedback mechanisms. Limited data are available to estimate the overall multi-organ injury incidence; two independent analyses described multi-organ irAEs occurring at a rate of roughly 5%, and a separate study found an incidence of 9% (14). They are generally manageable with high-dose glucocorticoids but can be fatal in some cases (30).

The multi-organ injuries of irAEs events in available case reports are shown in **Table 2** (30–40). All the patients with multiple organ injuries are male. The patient’s age ranged from 33 to 75 years, with an average age of 64 years old. Most complications occur within the first or second cycles of ICIs exposure. They are generally manageable with glucocorticoids and IVIG. As the first treatment, most of the patients were administered high-dose methylprednisolone (approximately 1-2 mg/kg/day). To our knowledge, only 5 cases of tislelizumab-induced multi-organ injuries have been reported to date (**Table 2**) (34–37, 39). Two are generally manageable with methylprednisolone, and the remaining three are manageable with methylprednisolone plus IVIG. Though the Society for Immunotherapy of Cancer (SITC) consensus definitions for irAEs terminology have been established for managing and treating individual organ irAEs, more experience should be needed in treating multiple-organs irAEs (14). In our case, the patient had multi-organ injuries after two cycles of tislelizumab. He also received methylprednisolone and IVIG, which is similar to that of previous reports. Therefore, effective management of severe irAEs from tislelizumab, including multi-organ injuries, can be partly achieved through methylprednisolone and immunoglobulins. Cases 5 and 6 described liver damage with only increased serum ALT and AST (34, 35). In contrast, our patient had severe hepatic damage with an increase of total bilirubin more than 14.5 times higher than the upper limit of normal. To our knowledge, such severe hepatic damage has not been reported. Case 6 reported kidney damage, characterized by an increase in creatinine levels up to 136 μmol/L. In contrast, our patient exhibited a creatinine increase to 432 μmol/L and required CRRT, indicating a more severe condition than that observed in Case 6. For instances of Grade 3 or 4 toxicity, defined as creatinine levels exceeding three times the baseline or greater than 4.0 mg/dL, inpatient hemodialysis should be considered, and steroid treatment initiated at a dosage of 1-2 mg/kg/day. Our patient experienced creatinine levels exceeding four times the baseline and underwent CRRT; such severe renal damage is uncommon.

**Table 2:** Multi-organ injuries of irAEs events in available case reports.

Case, year	Cancer type	Age (yr)	Sex	ICIs	IrAEs	Cycles of onset from ICIs	Treatment (dosage)	Outcome
Case 1, 2019 (30)	CHL	33	M	Nivolumab	Dermatitis, pancreatitis, hypothyroidism, myocarditis, rhabdomyolysis	8	GCs (1 to 2 mg/kg/d), IVIG, MMF	Dead
Case 2, 2019 (31)	Melanoma	70	M	Pembrolizumab	Dermatitis, vitiligo, autoimmune nephritis, autoimmune hepatitis, autoimmune encephalitis	4	GCs (high-dose), IVIG	Recovery
Case 3, 2020 (32)	Melanoma	70	M	Nivolumab, Ipilimumab	Colitis, diabetes, thyroiditis, hepatitis	1	MP (2 mg/kg/d), IFX	Recovery
Case 4, 2020 (33)	Melanoma	74	M	Pembrolizumab	Catastrophic antiphospholipid syndrome, multiple organ failure of lung, gastro-intestinal, renal, and liver	27	Prednisolone (60 mg/d)	Dead
Case 5, 2021 (34)	NSCLC	71	M	Tislelizumab	Lung, muscle, myocardium, liver, pituitary damage	1	MP (80 mg/d)	Recovery
Case 6, 2021 (35)	UUC	66	M	Tislelizumab	Myocarditis, myositis, liver and kidney damage	1	MP (1.5 mg/kg/d), IVIG	Recovery
Case 7, 2022 (36)	CC	65	M	Tislelizumab	Myasthenia gravis, myocarditis, rhabdomyolysis	1	MP (1 g/d), IVIG	Recovery
Case 8, 2022 (37)	LUSC	75	M	Tislelizumab	Toxic epidermal necrolysis, granulocytopenia	1	MP (NA), IVIG	Recovery
Case 9, 2023 (38)	Melanoma	60	M	Nivolumab, Ipilimumab	Diarrhea, pancytopenia, renal failure, hepatitis, myocarditis	1	MP (125 mg/d)	Recovery
Case 10, 2024 (39)	BCa	67	M	Tislelizumab	Adrenal hypofunction, Psoriasisby	2	MP (8 mg/d)	Recovery
Case 11, 2024 (40)	Melanoma	53	M	Nivolumab, Ipilimumab	Hypothyroidism, adrenal deficiency, acute interstitial nephritis	2	MP (1 mg/kg/d)	Recovery
This Case	LUSC	68	M	Tislelizumab	Striated muscle, liver, kidney, and thyroid damage	2	MP (1.5 mg/kg/d), IVIG	Recovery

IrAEs, immune-related adverse events; ICIs, immune checkpoint inhibitors; CHL, classic Hodgkin lymphoma; NSCLC, non-small cell lung cancer; UUC, ureteral urothelial cancer; CC, colon cancer; LUSC, lung squamous cell carcinoma; BCa, bladder cancer; M, male; GCs, glucocorticoids; MP, methylprednisolone; IVIG, intravenous immunoglobulin; MMF, mycophenolate mofetil; IFX, infliximab; NA, not mentioned.

All patients recovered except for two patients who died (**Table 2**). The correlation between multi-organ irAEs and survival outcomes has been studied. A multi-center study revealed that patients experiencing ICIs-associated myocarditis exhibited multi-organ irAEs, with a notably higher incidence of severe myocarditis, increased mortality, and poorer prognosis compared to patients with pure myocarditis (41). However, little is known about the specific prognosis of co-occurrence patterns of irAEs. Recently, Guihong Wan et al. conducted a retrospective multicohort study about the co-occurrence patterns of and survival outcomes after multi-organ irAEs among recipients of ICIs (42). Co-occurring irAEs were decomposed into seven factors across organs, including endocrine, cutaneous, respiratory, gastrointestinal, hepatic, musculoskeletal, and neurological. It found that patients dominated by endocrine and cutaneous irAEs were associated with improved survival at the 6-month landmark time point. At the same time, the other clusters either had unfavorable (respiratory) or neutral survival outcomes (gastrointestinal, musculoskeletal, hepatic, and neurological). These findings contribute to a deeper understanding of the potential biological mechanisms underlying irAEs across various organs.

As the clinical use of immunotherapy continues to increase, understanding and managing its unique toxicity becomes critical. The most urgent problem in clinical practice is minimizing the problems caused by irAEs while maximizing the therapeutic benefit of ICIs. It is also important to realize that more patients with irAEs of the treatment with ICIs will be admitted; multi-disciplinary consultation is essential because of the difficulty of early recognition and optimal treatment of these possible lethal side effects. Clinicians should fully understand the diversity and severity of adverse reactions to immunotherapy drugs, improve the ability to make early diagnoses and treatment, and pay attention to medication details so that these drugs can play a better role and bring more clinical benefits to patients.

In conclusion, ICI treatment may be a “double-edged sword.” Our patient presented with multiple organ damage, not single immunity treatment adverse reactions, relatively rare. In clinical work, irAEs are likely not a single-system organ disorder and many kinds of attention need to be combined with the risk of multi-system damage. More clinical data are still required to feature tislelizumab.

## Data Availability

The original contributions presented in the study are included in the article/supplementary material. Further inquiries can be directed to the corresponding author.
