# Corrigendum: Case report: Multi-organ injuries induced by tislelizumab

**DOI:** 10.3389/fimmu.2025.1630619

**Published:** 2025-06-11

**Authors:** Man Yuan, Ning Han, Li Shu, Libo Yan, Hong Tang

**Affiliations:** ^1^ Center of Infectious Diseases, West China Hospital of Sichuan University, Chengdu, China; ^2^ Division of Infectious Diseases, State Key Laboratory of Biotherapy, Sichuan University, Chengdu, China; ^3^ Department of Infectious Diseases, Suining Central Hospital, Suining, China

**Keywords:** lung squamous cell carcinoma, immune-related adverse events, immune checkpoint inhibitors, tislelizumab, multi-organ injuries, case report

In the published article, there was an error. A portion of the content in **Case description** is missing.

A correction has been made to **Case description**, Paragraph 1. This sentence previously stated:

“On evaluation at our institution, his body temperature was 36.5°C, with a pulse of 110 beats per minute, blood pressure of 98/72 mmHg, and a breath of 20 times/min. Hematology showed a hemoglobin level of 88 g/L, a white blood cell count of 7.06×10^9^/L, 89.2% neutrophils, and 6.9% lymphocytes. Chest CT revealed a small mass (1.4 x 0.9 cm) in the anterior lobe of the left superior lung, bronchial wall truncation, and peripheral pleural stretch (**Figure 1I**).”

The corrected sentence appears below:

“The patient was a 68-year-old man with a smoking history of 40 years. He had been diagnosed as having LUSC (cT3N0M0 IIB) by percutaneous needle lung biopsy in December 2023 at the local hospital. The pathology report showed P40 (+), pan-CK (AE1/AE3) (+), TTF-1 (-), NapsinA (-), CgA (-), CD56 (-), and Ki-67 (60% +) (**Figures 1A–F**). A contrast-enhanced chest computed tomography (CT) scan revealed a subpleural soft tissue density shadow in the anterior segment of the left upper lobe, with a maximum cross-sectional dimension of approximately 3.4 x 3.1 cm (**Figure 1G**). Then he had undergone neoadjuvant chemotherapy with paclitaxel (400 mg d1), carboplatin (400 mg d1), and tislelizumab (200 mg d1; BeiGene, China) Q3W in December 2023 at the local hospital. The first session of treatment went well, with no significant adverse effects. After the second dose of treatment in February 2024, he developed hyperthyroidism and then hypothyroidism and was given metoprolol succinate followed by levothyroxine. A follow-up CT scan showed significant reduction in the mass size (**Figure 1H**). In March 2024, he experienced nausea, poor appetite, and abnormal liver function markers and was admitted to West China Hospital of Sichuan University in April 2024. The time axis of diagnosis and treatment of the patient is shown in **Figure 2**. Since November 2023, he had been on atorvastatin calcium and aspirin for cerebral infarction without limb movement issues. He stopped atorvastatin in March 2024 due to liver function abnormalities.

On evaluation at our institution, his body temperature was 36.5°C, with a pulse of 110 beats per minute, blood pressure of 98/72 mmHg, and a breath of 20 times/min. Hematology showed a hemoglobin level of 88 g/L, a white blood cell count of 7.06×10^9^/L, 89.2% neutrophils, and 6.9% lymphocytes. Chest CT revealed a small mass (1.4 x 0.9 cm) in the anterior lobe of the left superior lung, bronchial wall truncation, and peripheral pleural stretch (**Figure 1I**).”

The authors apologize for this error and state that this does not change the scientific conclusions of the article in any way. The original article has been updated.

